# Relationship between handedness and joint involvement in rheumatoid arthritis

**DOI:** 10.1038/srep39180

**Published:** 2016-12-15

**Authors:** Ai Yaku, Motomu Hashimoto, Moritoshi Furu, Hiromu Ito, Noriyuki Yamakawa, Wataru Yamamoto, Takao Fujii, Fumihiko Matsuda, Tsuneyo Mimori, Chikashi Terao

**Affiliations:** 1 Department of Rheumatology and Clinical Immunology, Kyoto University Graduate School of Medicine, Kyoto, Japan; 2 Department of the Control for Rheumatic Diseases, Kyoto University Graduate School of Medicine, Kyoto, Japan; 3 Department of Orthopaedic Surgery, Kyoto University Graduate School of Medicine, Kyoto, Japan; 4 Department of Rheumatology, Kyoto-Katsura Hospital, Kyoto, Japan; 5 Department of Health Information Management, Kurashiki Sweet Hospital, Kurashiki, Japan; 6 Center for Genomic Medicine, Kyoto University Graduate School of Medicine, Kyoto University Graduate School of Medicine, Kyoto, Japan; 7 Center for the Promotion of Interdisciplinary Education and Research, Kyoto University Graduate School of Medicine, Kyoto, Japan; 8 Division of Rheumatology, Immunology, and Allergy, Harvard Medical School, Boston, MA 02115, USA; 9 Division of Genetics, Brigham and Women’s Hospital, Harvard Medical School, Boston, MA 02115, USA; 10 Program in Medical and Population Genetics, Broad Institute, Cambridge, MA 02142, USA

## Abstract

Rheumatoid arthritis (RA) is characterized by autoimmune chronic joint inflammation, which is worsened by mechanical stress. It is still inconclusive whether joints on the right side or the dominant side get more damaged in RA since the limited number of patients analyzed in the previous study had made it difficult to separately analyze right-handed and left-handed patients. Here, we enrolled 334 RA patients, the biggest number of patients in studies to address this issue and separately analyzed right-handed and left-handed patients. As a result, we observed that joints on the dominant side got clinically and radiologically more involved in the right-handed patients (p ≤ 0.0030). Importantly, this tendency was also seen in the left-handed patients, while it was not statistically significant due to the small sample size. This tendency was observed in each component of clinical or radiological involvement. Thus, handedness influences the laterality of clinical and radiological joint involvement in RA.

Rheumatoid arthritis (RA) is a chronic autoimmune disease that is characterized by synovitis and deformity of the joints^1^. Accumulation of inflammation causes joint destruction in patients with RA. Thus, it is important to control the inflammation in daily clinical practice and prevent joint destruction for the treatment of RA. In clinical practice we use the Disease Activity Score-28 (DAS28)^2^,^3^ and the modified Total Sharp Score (mTSS)^4^ or the Larsen method^5^,^6^ as measurements for disease activity and joint destruction, respectively.

DAS28 is composed of scores in the 28 joints that are examined. The DAS28 includes tender joint count (TJC), swollen joint count (SJC), patient global assessment (0 to 10 scale), physician global assessment (0 to 10 scale) and C-reactive protein level or erythrocyte sedimentation rate. In mTSS, hand and foot radiographs are used for scoring the erosion scores in 16 joints of hands and wrists (graded from 0 to 5), and in 6 joints of the feet (graded from 0 to 10), and joint space narrowing (JSN) scores in 15 joints of the hands and wrists (graded from 0 to 4) and in 6 joints of the feet (graded from 0 to 4)^4^. In the Larsen method a score (0–5) is assigned to each of the distal interphalangeal (DIP), proximal interphalangeal (PIP), metacarpophalangeal (MCP) joints, the interphalangeal joint of hallux and the second to fifth metatarsophalangeal (MTP) joints. In the Larsen method, the wrist is considered as one unit and the score for the wrist is multiplied by five^5^,^6^. mTSS provides separate scores for erosion and for JSN, whereas the Larsen method includes both erosions and joint space narrowing in each joint as a single score. Recently mTSS tends to be used more widely than the Larsen method, partly because some studies say that mTSS is more precise and reliable compared to the Larsen method^7^.

Inflammation in RA patients is decreased not only by drugs but also by resting or immobilization of the affected joints. Peter Lee *et al*. (1974) compared RA patients between out-patient treatment group and in-patient treatment group. They reported in-patient treatment group with at least 13 hours bed rest improved pain severity, duration and severity of morning stiffness, articular index of joint tenderness and grip strength^8^. Furthermore, some reports described the influence of motor laterality on RA^9^,^10^. When patients with hemiplegia develop RA, the arthritis of the paralyzed side developed less severely than the other side^11^,^12^,^13^. Thus mechanical stress plays an important role on inflammation of RA.

Mechanical burden may worsen joint inflammation, and the dominant hand joints would be affected more than the non-dominant joints. There are multiple previous studies which tried to address this issue^14^,^15^,^16^,^17^,^18^,^19^,^20^,^21^,^22^. However, they analyzed a relatively small number of patients with RA and evaluated only the right-handedness or did not evaluate the joint involvement differently in right-handed and left-handed patients. Thus, it is still unclear whether right-side joints or dominant-side joints get more affected in patients with RA. Furthermore, most of the previous studies evaluated either disease activity or joint destruction in the patients. Many studies evaluating joint destruction used not mTSS but the Larsen method. To clarify the relationship between handedness and joint involvement of RA in detail, here we performed the biggest study ever and separately analyzed the right-handed and the left-handed patients with RA by using both measurements for disease activity and joint destruction, namely, DAS28 and mTSS.

## Results

To confirm the primary aim of this study, we at first searched for previous reports which addressed the relationship between handedness and joint involvement of RA. We chose six reports^14^,^15^,^16^,^17^,^20^,^21^ and an additional three reports referred to in the six reports (for detail, see Materials and Methods)^18^,^19^,^22^. Thus, a total of nine previous reports were identified (Table 1). The maximum number of patients was 292 in the previous reports^22^. Six reports listed the information of handedness^14^,^15^,^16^,^17^,^18^,^19^ but four reports only examined the right-handed paients^14^,^15^,^18^,^19^. Two reports examined the right-handed and left-handed patients although they did not evaluate them separately^16^,^17^. The joint symptoms were used as a measurement in one report^17^ and X-rays were used in eight reports^14^,^15^,^16^,^17^,^18^,^19^,^20^,^22^. Only two out of the eight reports used mTSS as evaluation of X-rays^16^,^18^. No previous reports used both the disease activity and X-rays. Based on the results of the studies, many of the previous reports concluded that the symptoms or joint destructions on the dominant sides were worse than those of non-dominant sides. However, none of them evaluated whether the joint damage on the left side was worse than that on the right side in left-handed patients (Table 1).

**Figure 1:**
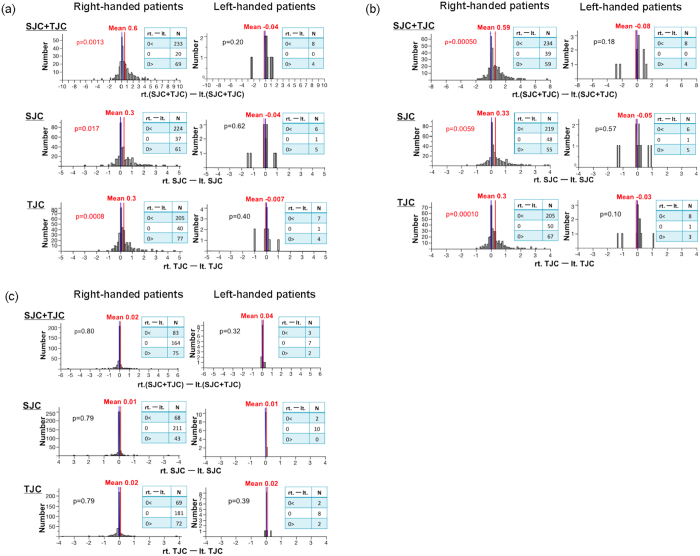
More clinical joint involvement on the dominant side in patients with RA. The difference in SJC and TJC, jointly or separately, between the two sides is shown for right-handed and left-handed patients. The red line indicates the mean value. The blue line shows the zero point. The score differences were assessed by Student t-test. X-axis: The score differences between the two sides (the right-side score minus the left-side score). Y-axis: The number of patients. (**a**) Upper + Lower extremities (**b**) Upper extremities (**c**) Lower extremities.

We recruited a total of 334 patients with RA in this study. The number of right-handed patients was 322 (96%) and the left-handed was 12 (4%) (Table 2). We evaluated the laterality of the daily disease activity using DAS28 and its components SJC and TJC (for details, see Materials and Methods). We observed that the dominant side in the right-handed patients showed significantly higher scores in the sum of SJC and TJC than the non-dominant side (p = 0.0013). We also observed this tendency in the left-handed patients, although the difference did not reach the statistically significant level (Fig. 1a). These may suggest that the dominant side, and not the right side in general, gets more involved compared to the non-dominant side in RA. When we also separately assessed SJC and TJC, the dominant side in the right-handed patients showed significantly higher scores than the non-dominant side both for SJC and TJC (p = 0.017 and p = 0.0008, respectively, Fig. 1a). Again, this tendency was also seen in the left-handed patients (Fig. 1a). When we further separately analyzed the upper and lower extremities, we observed that the more involvement of the dominant side in right-handed patients was mainly driven by the upper extremities (for SJC + TJC, SJC and TJC, p = 0.00050, 0.0059 and 0.00010 in upper extremities, respectively, Fig. 1b and p = 0.02, 0.79 and 0.79 in lower extremities, respectively, Fig. 1c). This tendency was also true in left-handed patients (Fig. 1b and c).

**Figure 2:**
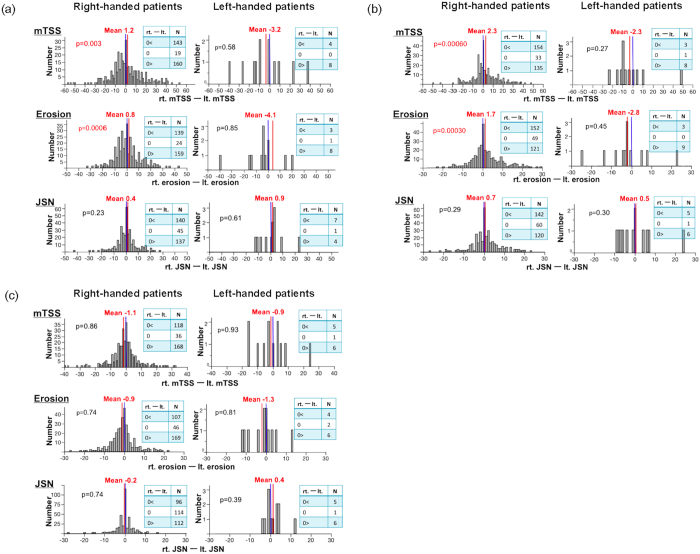
More radiological joint involvement on the dominant side in patients with RA. The difference in erosion and JSN for mTSS, jointly or separately, between the two sides is shown for right-handed and left-handed patients. The blue line shows the zero point. The score differences were assessed by Student t-test. X-axis: The differences in mTSS or its component between the two sides (the right-side score minus the left-side score). Y-axis: The number of patients. The red line indicates the mean value. (**a**) Upper + Lower extremities (**b**) Upper extremities (**c**) Lower extremities.

Since accumulation of the daily disease activities leads to joint destruction in patients with RA, a more severe involvement of joints on the dominant side would lead to severe joint destruction in the same side. Thus, next we evaluated the laterality of mTSS. We observed that right-handed patients demonstrated a more severe joint destruction on the dominant side (p = 0.0030, Fig. 2a). When we analyzed mTSS in detail, we found that the severe joint destruction on the dominant side was mainly driven by erosion (p = 0.00060, Fig. 2a). This tendency of severe joint destruction on the dominant side was also seen in the left-handed patients (Fig. 2a). When we separately examined the laterality in the upper extremities and lower extremities, we found that the difference on the dominant side was clear in the upper extremities (mTSS and erosion, p = 0.00060 and 0.00030, respectively, for mTSS and erosion, Fig. 2b). This was also true for left-handed patients (Fig. 2b). mTSS and erosion in the lower extremities did not show a clear difference between the two sides, especially in the right-handed patients (p = 0.85 and 0.74, respectively, for mTSS and erosion, Fig. 2c).

Since the current study contained a limited number of left-handed subjects, there was not enough power to obtain statistical significance for analyses using only left-handed subjects in spite of the tendency for a more severe involvement in the dominant side. However, when we compared score differences in mTSS of upper-extremities, the main driver of laterality in mTSS, between the right-handed and the left-handed, we obtained significant difference (p = 0.040), suggesting different laterality between the two groups.

**Figure 3:**
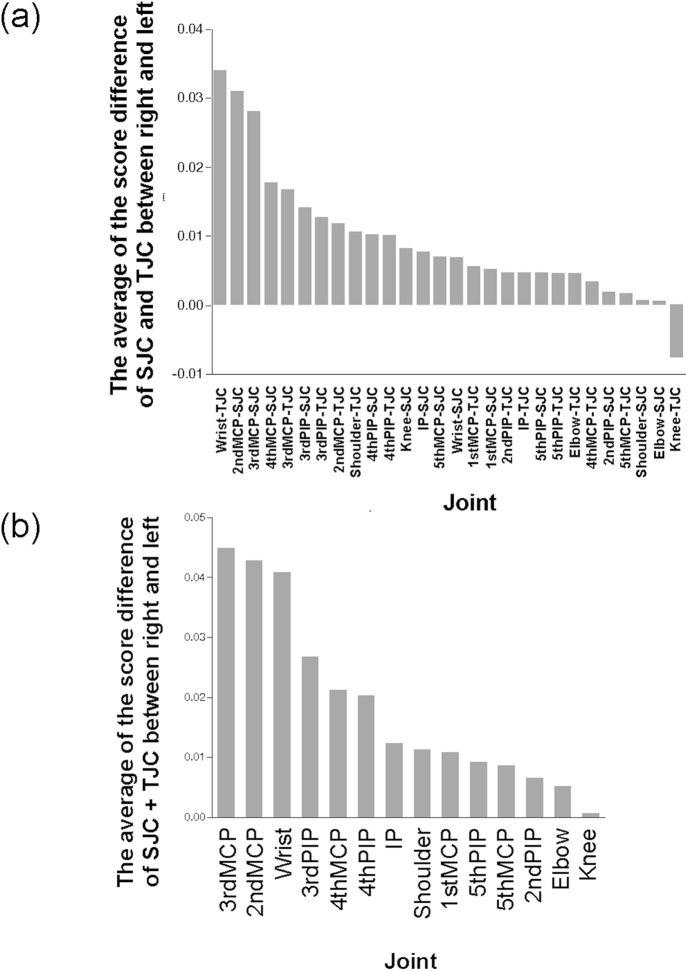
More clinical joint involvement on the dominant side in patients with RA regardless of joints except for knee. The difference in SJC and TJC, separately (**a**) or jointly (**b**), between the two sides is shown for each joint in the right-handed patients with RA. IP = Interphalangeal.

Finally, we separately evaluated each joint to analyze whether there were joint-specific tendencies. Since this was a very fine subdivision of joints which required a substantial number of subjects for statistical power, we analyzed only right-handed patients considering the small number of left-handed patients. When we analyzed TJC and SJC separately, we found that TJC of the knee was the only exception showing a more severe non dominant-side involvement (Fig. 3a). When we analyzed sum of TJC and SJC, all of the joints showed a more severe dominant-side involvement while the knee showed just a slight difference (Fig. 3b). These suggest that there is a consistent mechanism underlying joint involvement across the joints evaluated in the upper extremities.

**Figure 4:**
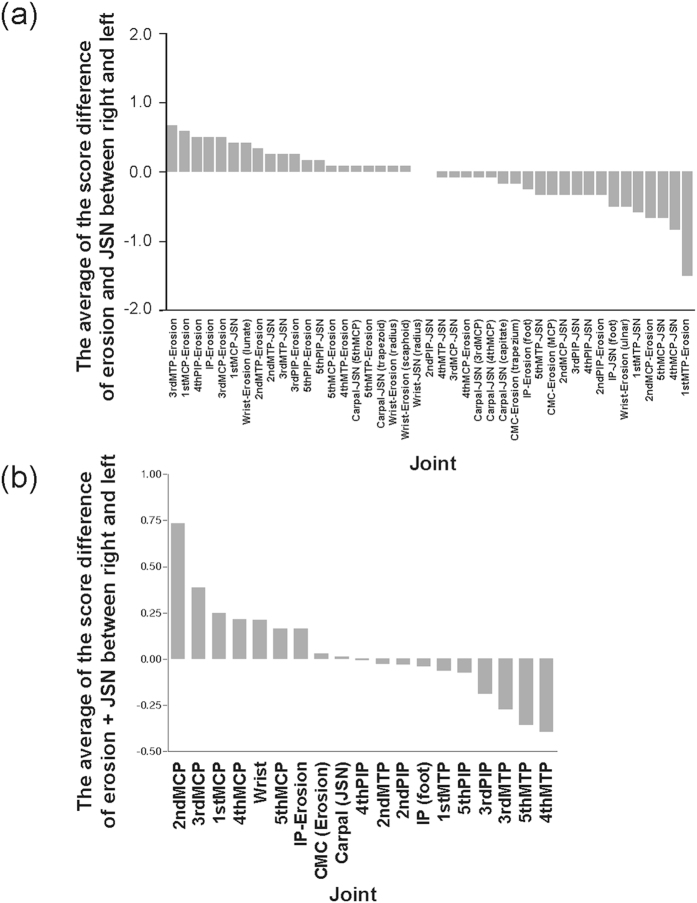
Possible difference in laterality of radiological involvement between the upper and lower extremities. The difference in erosion and JSN as mTSS components, seprarately (**a**) or jointly (**b**), between the two sides is shown for each joint in the right-handed patients with RA.

When we assessed mTSS, we found that MTP joints showed a more severe non dominant-side destruction (Fig. 4). MCP joints especially the 2^nd^ and 3^rd^ MCP showed the biggest difference in both DAS28 and mTSS between the two sides (Figs 3b and 4).

## Discussion

Previous studies have reported the laterality of RA with handedness. However, all of them led to the conclusion by evaluating only the right-handed patients, or without evaluating the left-handed patients separately from the right-handed patients. Thus, there might be a possibility that the right joints were generally worse than the left joints regardless of handedness. This is the first study looking at the laterality of joint involvements, evaluating the left-handed patients separately from the right-handed patients, and using both SJC/TJC and mTSS. Further, this is the largest study ever to address the relationship between handedness and joint involvement in RA.

There were no differences in baseline characteristics between the right-handed and left-handed groups, although the power was limited due to the small number of the left-handed patients.

The natural rate of left-handedness is generally around 10%^23^,^24^,^25^, In our study the rate of left-handed patients was 4%. It may be notable that the ratio of the left-handed in the two previous studies including left-handed patients were comparable to the current study (3.2 and 4.6%)^16^,^17^. The discrepancy of rate of left-handedness between RA and the general population might be explained by male-female ratio in RA. The previous study reported that males are more prone to be left-handed than females^26^. Thus, 87% female patients with RA in the current study and the comparable ratio in the previous study could lead to the low rate of left-handedness.

There was a more clinical joint involvement on the dominant side evaluated by DAS28 in the right-handed patients. mTSS in the dominant side joints were also worse than those in the non-dominant ones. These results confirmed that the accumulation of clinical involvement led to radiological joint destruction in RA. While both erosion and JSN showed this tendency, the difference was mainly driven by erosion. The tendency of laterality in both DAS28 and mTSS was also seen in the left -handed patients, although it was not statistically significant likely due to the small sample size. This study is still under-powered to obtain statistical significance in left-handed patients alone. We need to recruit more patients (especially the left-handed) to address this point. However, significant difference in score difference of mTSS between the left-handed and the right-handed supports the difference in laterality of joint involvement between the two groups.

Dickson *et al*. reported the index and middle fingers had significantly stronger flexion and pinch force than the ring and little fingers^27^. Mody GM *et al*. showed that the index and middle fingers were severely affected in RA patients^15^. Kawabata *et al*. showed significant differences between pinch strength in the dominant and non-dominant hands were found for the thumb-index finger and thumb-middle finger, with the dominant hand having 18% greater strength^28^. Our results showed that the 2^nd^ and 3^rd^ MCP joints on the right side of the right-handed patients were most severely involved both in SJC and TJC, and erosion and JSN. This seems to agree with the previous studies and may suggest that the index and middle fingers were used more often than the other fingers in daily life.

It would be notable that the current study showed possible difference in laterality, especially for mTSS, between upper and lower extremities. This difference seemed to be driven by MTP joints which were not evaluated by DAS28. Many of the right-handed patients are right-footers and use the left foot to keep balance^29^. Hatta *et al*. evaluated the relationship between the upper- and lower-limbs laterality and the difference in kinesthetic function between the lower limbs. The muscle of the left lower limb in the right-handed patients was stronger than that of the right lower limbs in the 50 s age group, but not in the other groups (30 s–40 s, 60 s and 70 s). This suggests that the muscle system of the left leg in right-footers may develop more than that of the right leg. Further expansion of subjects in future studies would lead to clear differences in laterality between the upper and lower extremities.

While statistical significance was obtained in the current study, this does not provide information about the effect size or the clinical significance. Since the difference in SJC and TJC between dominant and non-dominant sides are less than one, this difference is not sufficient to give clinical advice to rheumatologists only to see the dominant-side of the patients with RA in the clinical practice. In future studies, we may characterize patients showing a more severe disease activity and joint involvement on the dominant side than the non-dominant side based on potential correlates such as occupations and may apply the results in clinical settings.

In conclusion, the current study indicates that joints on the dominant side in the right-handed are clinically and radiologically more affected compared to the non-dominant side in patients with RA. Although the power was small, the same tendency was seen in the left-handed.

This study also may suggest potential difference in laterality between the upper and lower extremities. Further replication studies would confirm the current results and help elucidate the detailed relationship between mechanical stress and joint involvement in RA.

## Materials and Methods

### Previous studies

To search for previous reports on the relationship between handedness and joint involvement of RA, we used PubMed with the following key words; “handedness” and “rheumatoid arthritis” on 28^th^ May 2016. The hit number was 52. We chose six reports that included “handedness” or “dominant hand” in the title^14^,^16^,^17^,^20^,^21^,^30^. The other 46 reports were excluded from the following analyses since they were not directly about the association between handedness and symptoms or outcomes in RA. From the references of these six reports we selected three more reports about handedness and rheumatoid arthritis^18^,^19^,^22^.

### Patients

We enrolled a total of 334 RA patients from the KURAMA (Kyoto University Rheumatoid Arthritis Management Alliance) cohort^31^. The KURAMA cohort was established in 2011 at the Center for Rheumatic Diseases in Kyoto University Hospital for tight control of RA and to utilize their sequential clinical and laboratory data for clinical investigations^31^. All of the patients with written informed consent fulfilled the revised 1987 ACR criteria for RA^32^ or the 2010 ACR/EULAR classification criteria for RA^33^. This study was designed in accordance with the Declaration of Helsinki and was approved by the Medical Ethics Committee of Kyoto University Graduate School and Faculty of Medicine before starting the study.

### Evaluations

Handedness was surveyed by a patient-based questionnaire. We used SJC and TJC to assess clinical joint involvement, and mTSS to evaluate joint destruction as radiological joint involvement and the accumulation of the inflammation. Rheumatologists routinely evaluated swelling and tenderness of the 28 joints evaluated for DAS28 in patients with RA on each office visit. Since each patient had different numbers of office visits, we took the average of SJC and TJC across the study period (from April 2011 to March 2015) in each patient. X-rays of hands and feet were taken in 2012. A trained rheumatologist (M. F.) using mTSS assessed the radiographs with intra-observer agreement of 0.93. The reader for mTSS and rheumatologists for TJC and SJC were blinded to the purpose of this study as well as patients’ information including handedness.

### Statistical analysis

We conducted all statistical analyses by using the JMP Pro 12.0.1 software program (SAS Institute Inc., Cary, NC, USA). We divided the patients into two groups according to their handedness. We used chi-square test or Student t-test to compare the characteristics between the right-handed and the left-handed patients. To estimate the laterality of joint involvement, we calculated the average of the score differences between the right and left sides (the right-side score minus the left-side score) and assessed whether the absolute value of the positive and negative score differences were different from each other by the Student t-test. We evaluated these score differences of clinical involvement (SJC + TJC) and radiological involvement (mTSS). We also analyzed SJC, TJC, erosion and JSN separately. We further separated these scores into upper and lower extremities. To show substantial difference in laterality of joint involvement between the right-handed and left-handed, we also compared the score differences in mTSS in upper extremities, the variable with the strongest laterality, between the right-handed and the left-handed by the Wilcoxon rank sum test.

We also evaluated the laterality in each joint. Since full scores of mTSS were not equally distributed to each joint, we introduced the following transformation in Fig. 4. The wrist score consisted of the average of three erosion score and one JSN score. The carpal score consisted of doubled average of the five JSN scores. The carpometacarpal score was the sum of two erosion scores. P-values less than 0.05 were regarded as statistically significant.

## Limitations

This study has several limitations. First, in spite of the biggest number in subjects analyzed, the number of the left-handed patients was still not enough to achieve statistical power. Second is the definition of handedness. We interviewed patients about handedness without using rating scales of handedness, e.g. the Edinburgh Handedness Inventory^34^. Self-reported handedness might have included the both-handed in the left-handed.

## Additional Information

**How to cite this article**: Yaku, A. *et al*. Relationship between handedness and joint involvement in rheumatoid arthritis. *Sci. Rep.* **6**, 39180; doi: 10.1038/srep39180 (2016).

**Publisher's note:** Springer Nature remains neutral with regard to jurisdictional claims in published maps and institutional affiliations.

## Figures and Tables

**Table 1:** Previous reports about laterality of joint involvement in patients with RA.

Author	Year	Journal	No.	Rt/Lt-handed	Score	Result
Vainio K	1953	Ann Rheum dis	292	Not listed	Ulnar deviation	Larger angles of dominant hands in females
Kemble JVH	1977	The Hand	61	Rt-handed	The degree of ulnar drift and erosion	More severe in right hands
Mattingly PC	1979	Rheumatology and Rehabilitation	30	Rt-handed	Sharp score	More severe in right hands
Owsianik WDJ	1980	Ann Rheum dis	20	Rt-handed	Larsen score	More severe in right hands
Hasselkus RB	1981	The American Journal of Occupational Therapy	51	Not listed	Lateral laxity and hyperextension loss of the MCP joints	No significant difference between dominant and nondominant hands
Mody GM	1989	Ann Rheum dis	233	Rt-handed	Larsen score	More severe in right hands
Boonsaner K	1992	Br J Rheumatol	93	Rt-handed:90 Lt-handed:3	Swelling and tenderness	More severe in dominant side
Pfeil A	2009	Rheumatol Int	128	Not listed	Joint space narrowing	More severe in right hands, but there was no significant difference
Koh JH	2015	Plos One	194	Rt-handed:185 Lt-handed:9	van der Heijde-modified Sharp Score method	More severe in dominant side

Ann Rheum dis = Annals of the Rheumatic Disease; Br J Rheumatol = British Journal of Rheumatology.

**Table 2:** Baseline characteristics.

	The right-handed (n = 322, 96%)	The left-handed (n = 12, 4%)	P value
Female, n (%)	281 (87)	10 (83)	0.7
Age, yr (SD)	63.6 (12.7)	62.5 (10.4)	0.8
Disease duration, yr (SD)	14.6 (11.7)	12.8 (10.1)	0.6
Stage I/II/III/IV, n	48/83/53/138	1/4/2/5	0.9
Class I/II/III/IV, n	80/182/57/3	1/10/1/0	0.3
Rheumatoid factor, IU/ml (SD)	86.0 (141.1)	63.8 (74.5)	0.6
Anti-citrullinated peptide antibody, U/ml (SD)	120.5 (112.7)	92.7 (107.6)	0.4
CRP (SD)	0.6 (1.1)	1.2 (3.3)	0.1
ESR, mm/hr (SD)	27.8 (22.5)	22.4 (14.9)	0.4
DAS28CRP (SD)	2.5 (1.1)	2.7 (1.1)	0.7
DAS28ESR (SD)	3.2 (1.2)	3.2 (1.1)	1
Methotrexate use, n (%)	230 (71)	8 (67)	0.7
Methotrexate dose, mg (SD)	5.2 (4.2)	4.7 (4.3)	0.7
Biologics use, n (%)	97 (30)	4 (33)	0.8

CRP = C reactive protein; ESR = Erythrocyte sedimentation rate ^*^Student-t test.
